# Individual Differences in Infants’ Temperament Affect Face Processing

**DOI:** 10.3390/brainsci10080474

**Published:** 2020-07-23

**Authors:** Jennifer L. Rennels, Andrea J. Kayl, Kirsty M. Kulhanek

**Affiliations:** Department of Psychology, University of Nevada, Las Vegas, NV 89154, USA; kayla@unlv.nevada.edu (A.J.K.); kulhanek@unlv.nevada.edu (K.M.K.)

**Keywords:** face perception, infancy, face discrimination, eye-tracking, visual search, emotional expression, race, gender, surgency, orienting

## Abstract

Infants show an advantage in processing female and familiar race faces, but the effect sizes are often small, suggesting individual differences in their discrimination abilities. This research assessed whether differences in 6–10-month-olds’ temperament (surgency and orienting) predicted how they scanned individual faces varying in race and gender during familiarization and whether and how long it took them to locate the face during a visual search task. This study also examined whether infants viewing faces posing pleasant relative to neutral expressions would facilitate their discrimination of male and unfamiliar race faces. Results showed that infants’ surgency on its own or in conjunction with their orienting regularly interacted with facial characteristics to predict their scanning and location of faces. Furthermore, infants’ scanning patterns (dwell times and internal–external fixation shifts) correlated with their ability and time to locate a familiarized face. Moreover, infants who viewed faces with pleasant expressions showed better discrimination of unfamiliar race and male faces compared with infants who viewed neutral faces. Including temperament in the analyses consistently demonstrated its significance for understanding infant face processing. Findings suggest that positive interactions with other-race individuals and men might reduce processing disadvantages for those face types. Locating familiar adults in a timely manner is a crucial skill for infants to develop and these data elucidate factors influencing this ability.

## 1. Introduction

Over the last two decades, infant face processing researchers have generated substantial data demonstrating how infants’ social experience is related to their face recognition abilities. For example, infants ranging in age from 3 to 16 months show more difficulty discriminating among exemplars of men’s faces than women’s faces [1,2,3,4]. Similarly, race familiarity affects infant face processing. Some studies have found 3-month-olds are poorer at discriminating between faces from unfamiliar than familiar racial groups [5,6], whereas other studies suggest that differences in recognition abilities based on racial familiarity become particularly evident during the second half of the first year [7,8]. These discrepancies in face processing are related to the predominant experience with a female primary caregiver and other women and individuals who are the same race as the primary caregiver [8,9,10]. Although findings on face processing advantages for familiar face types are consistent, the effect sizes are typically small, suggesting a variety of factors, including individual differences among infants, may influence their discrimination abilities [11]. The current study therefore examined individual differences in infants’ temperament and whether these differences were related to their facial scanning and perceptual expertise.

Temperament refers to variations in how individuals react and regulate their responses, and manifests as a function of both genetic and environmental influences [12,13]. It affects infants’ reactions to novel and familiar stimuli [12], so it is plausible that measures of temperament are related to how infants respond to female and male faces from familiar and unfamiliar races. Although one study found no relation between 3-month-old Caucasian infants’ temperament and their recognition of Caucasian and African female faces, they also did not find any mean differences in the infants’ ability to recognize the Caucasian and African faces [14]. It might be necessary to test these effects later in development. Indeed, research suggests that if infants develop perceptual expertise for certain face types or expressions, it typically manifests during the latter part of the first year [7,8,15,16]. Temperament is also thought to emerge across the first year of life [13,17]. Moreover, research that has found significant relations between infant temperament and their visual or neural attention toward faces varying in emotional expression has included infant samples with mean ages of 7–12 months [18,19,20,21,22]. As such, we tested infants aged 6 to 10 months.

We assessed how orienting/regulatory capacity (henceforth referred to as orienting) and positive affectivity/surgency (henceforth referred to as surgency) predicted infants’ looking behavior while being familiarized to a face, their ability to locate the face and the time it took to locate the face during a visual search task [2]. We also examined correlations between the familiarization behaviors and face location variables to investigate what best predicted face recognition/location abilities. Orienting encapsulates behaviors such as duration of orienting, cuddliness, soothability and low intensity pleasure. Surgency encompasses behaviors such as approach, vocal reactivity, smiling and laughter, activity level, perceptual sensitivity and high-intensity pleasure [23]. Higher levels of infant orienting and surgency positively predicted their effortful control in toddlerhood [24] and infants’ surgency at 10 months positively correlated with their sustained attention at 10 months and their global executive functioning at 18 months [25]. Although high surgency at 10 months positively correlated with infants’ cognitive regulation at 10 months, it negatively correlated with their emotion regulation at 18 months [25]. Based on these data, we predicted that infants high in orienting would display facial scanning behaviors during familiarization trials that positively correlated with successfully locating the face and negatively related with the time to locate the face (i.e., quicker at finding the face). As such, they should display better face recognition. Our hypotheses regarding surgency were more exploratory because both cognitive and emotional regulation could affect infants’ performance during the study.

Furthermore, there is limited research on the relation between infant temperament and looking behavior in relation to face recognition, but there is evidence that temperament affects their attention toward faces neurally and behaviorally. For example, infants high in one component of surgency (smiling and laughter) who also had mothers with a high positive disposition demonstrated a stronger response to fearful than happy faces than those low in smiling and laughter as evidenced by a larger Nc amplitude, an indicator of attentional orienting or arousal [19]. Similarly, infants high in orienting showed a larger Nc amplitude and quicker responding to fearful faces compared with those low in orienting [20]. Temperament has also been found to correlate with infants’ looking behavior. The average fixation duration of infants aged 4–10 months while viewing videos of dynamic faces, flashing and moving shapes and multimodal objects positively predicted their effortful control and negatively predicted their surgency and hyperactivity-inattention at 19–36 months of age [26]. Albeit limited, data suggest high levels of orienting should be related to infants’ face processing expertise [20] but the data regarding infant surgency are again mixed. A component of high surgency correlated with a neural response indicative of attention [19], whereas low surgency was related to longer fixation durations, a potential indicator of the encoding process [26]. Therefore, we were uncertain whether high or low levels of surgency would best predict scanning behaviors during familiarization that correlated with face recognition.

Infants’ attention during familiarization might also be related to the facial expression posed by the model. Most studies examining infants’ recognition of familiar and unfamiliar face types have utilized images with neutral expressions [2,3,4,6,7,8], but most infants do not typically experience neutral faces and tend to respond negatively to such faces as in the still-face paradigm [27]. Four- to 6-month-olds look longer at smiling than neutral faces and typically learn to categorize happy emotions first, likely due to most infants’ familiarity with positive expressions [28,29], so infants might scan and process pleasant and neutral expression faces differently. Indeed, 3- and 7-month-olds’ recognition of faces was enhanced when they were familiarized to faces posing smiles as opposed to a neutral expression [30,31]. Moreover, when familiarized to and tested with faces posing happy expressions, White 6-month-olds recognized unfamiliar race (Asian) female faces and White 9-month-olds recognized unfamiliar race (Black) female faces. In contrast, they did not show such discrimination when the same faces posed neutral expressions [32].

Neutral expressions can be emotionally ambiguous—adults judged neutral faces as posing various emotions, such as tired, concerned or mildly surprised [33]. Such ambiguity combined with less experience with unfamiliar race faces and male faces might contribute to infants’ difficulty in discriminating these faces. Moreover, males’ neutral expressions are more structurally similar to angry expressions than are females’ neutral expressions [34], which might make neutral male faces seem threatening. If so, it could potentially affect facial scanning behavior and hinder face recognition. 

It should be noted, however, that in Quinn et al.’s study [32], White 6-month-olds recognized unfamiliar race (Asian) female faces and White 9-month-olds recognized unfamiliar race (Black) female faces when familiarized to and tested with the faces posing angry expressions. Angry female faces therefore did not hinder infants’ face recognition but rather enhanced it, similar to the faces posing happy expressions [32]. These effects were not due to longer looking during familiarization or greater discriminability of the emotionally paired versus neutrally paired faces. Rather, the authors suggested the facial emotions led to infants processing the faces at a deeper level, with happy expressions offering potential social connection and angry expressions conveying potential danger. Subsequently, infants might have been motivated to individuate the emotional faces [32] and potentially engaged in different scanning behaviors when familiarized to faces with neutral versus emotional expressions. As such, the current investigation assessed whether scanning behaviors differed between infants in conditions where they viewed familiar and unfamiliar race faces posing either pleasant or neutral expressions during familiarization. Moreover, we examined whether these findings generalized to familiarization of dynamic faces and male faces because the faces in Quinn et al.’s study were static images of women [32]. Last, if the potential for social connection manifests when infants view pleasant relative to neutral expression faces, orienting and surgency (which are related to experiencing pleasure based on stimulus intensity and novelty [24]) should interact with facial expression and race to affect infants’ scanning of the familiarized face.

During familiarization, we assessed infants’ total dwell time (i.e., fixations and saccades) toward the whole face and the dwell time and number of fixations infants made to the internal and external facial features. These scanning behaviors should be informative regarding whether and how infant temperament interacts with facial cues during the encoding process even though others did not find differences in 6-and 9-month-olds’ looking time toward static images of neutral or happy female faces [32], or 7-month-olds’ dwell time and number of internal feature fixations toward static images of happy and neutral female faces [35]. Since infants have a predominant experience with female faces [9,10] and their varied emotional states, they may respond similarly to neutral and happy female faces. Infants have less experience with male faces [9,10], however, so they might attend to their faces differently as a function of expression and/or race, so we explored this possibility in our study. Moreover, dynamic images conveying neutral versus pleasant expressions might produce different attentional patterns compared with static images.

We also investigated the number of fixation shifts infants made between the internal facial features (internal–internal fixation shifts), and between the internal and external facial features (internal–external fixation shifts) during familiarization. Such shifts should be indicative of second-order relational and holistic processing, which facilitate face encoding and processing of emotional expression [36,37,38]. Furthermore, data demonstrate that internal–internal feature shifts are related to infants’ processing of familiar race faces. For example, monoracial 3-month-olds’ visual shifts between the eye and mouth region while habituating to a static image of a familiar race face correlated with their ability to distinguish between a familiar and novel face at test [39]. Moreover, the number of fixation shifts that 6-and 9-month-olds made between the eyes, nose and mouth of a dynamic face during familiarization facilitated their ability to discriminate between the familiar and a novel face [40].

To summarize our hypotheses, we predicted that infant orienting and surgency would interact with facial cues (expression, race and gender) to affect scanning behaviors during familiarization. We expected high orienting to predict scanning behaviors during familiarization that correlated with ability to locate the face and time to locate the face. We also expected high orienting to predict a higher number of faces located and a quicker time to locate the face. We were uncertain as to whether high or low surgency would function in a manner similar to high orienting given the mixed results from the limited prior research. Viewing positive relative to neutral expressions should affect infants’ scanning behaviors during familiarization and facilitate infants’ recognition of male and unfamiliar race faces. We expected number of fixation shifts (both internal–internal and internal–external) to positively correlate with ability to locate the face [38,39,40] and negatively correlate with time to locate the face. We also explored whether longer dwell time and number of fixations, particularly those toward the internal facial features, positively predicted face location and negatively predicted location time.

## 2. Materials and Methods

**Participants.** Infants (*N* = 108, 47 girls) aged 6 (*n* = 15, *M* = 186.27 days; *SD* = 8.36), 7 (*n* = 21, *M* = 215.62 days; *SD* = 7.94), 8 (*n* = 22, *M* = 244.09 days; *SD* = 8.30), 9 (*n* = 25, *M* = 277.364 days; *SD* = 7.05) and 10 months (*n* = 25, *M* = 308.32 days; *SD* = 10.70) were included in the analyses. Most infants had a female primary caregiver (*n* = 98), but eight infants had a male primary caregiver and two infants had parents share primary caregiving. The race/ethnicity of the primary caregivers was: White (*n* = 66); Mexican/Mexican American/Chicano/Spanish/Hispanic/Latino (*n* = 27); White/Asian (*n* = 7); Black/African American (*n* = 5); Asian/Asian American (*n* = 2); and one family did not identify the caregiver’s race/ethnicity. An additional 145 infants (79 girls) participated but did not have any data included in the analyses because they did not meet our inclusion criteria of a minimum of 1 s looking to the face during the familiarization phase and/or minimum of 800 ms of looking to the screen during the search phase for any trials (*n* = 26), or were fussy (*n* = 36), preterm/developmentally delayed (*n* = 22), missing temperament data (*n* = 6) or off task (*n* = 4), or due to data loss on all trials (typically due to poor calibration, inaccurate or inconsistent crosshair or baby repositioning) (*n* = 24), or the experimenter was unable to calibrate eye movements (*n* = 17), or an equipment (*n* = 9) or experimenter error (*n* = 1) occurred. To recruit families with infants eligible to participate, we utilized marketing lists and social media. 

**Facial Experience.** To measure infants’ experience with female and male faces and Black and White faces, we had their parents complete the Infant-Individual Interaction Scale (IIIS) and Infant-Caregiver and Family Member Interaction Scale (ICFMIS; [9]) every day for approximately one week prior to the study. The IIIS enables parents to document their infant’s interactions with infrequently encountered individuals or strangers and provides a place for them to note the interacting person’s demographics (approximate age, gender and race), length of interaction and their infant’s attention toward the person. The ICFMIS permits parents a more efficient way to indicate how often their infant interacted with frequently experienced individuals and whether each interaction entailed a high, moderate, brief or fleeting level of involvement. These scales are reliable, show construct validity [9] and provide data regarding percent experience with certain face types similar to those from cameras mounted on an infant’s head to record interactions [10]. 

**Temperament.** Parents completed the Infant Behavior Questionnaire-Revised (IBQ-R) very short form, which includes 37 items and is appropriate for assessing 3-to 12-month-olds’ surgency and orienting [23]. The IBQ-R also assesses infants’ negative affect but that measure was not included in this study—preliminary analyses for another research report showed that infants high in negative affect were less likely to complete the visual search task, thus skewing the distribution of these data. Parents also completed surveys regarding their infants’ facial experience [9] and motor development (those data are not included in this report) [41] on a daily basis, so we utilized the very short form of the IBQ-R to reduce the burden on their time. Putnam et al. demonstrated that this shortened scale was both reliable and valid across diverse samples of infants [23]. 

**Apparatus.** To measure infants’ eye movements, we used an Applied Science Laboratories D6 desktop remote eye tracker with the model 6000 control unit. The D6 optics module and camera tracks at 60 Hz and utilizes face recognition software to compensate for head movements within approximately 0.093 square meters. It has an accuracy of 0.5° with a tracking range of 50° horizontal and 40° vertical [42]. Stimuli displayed centered on a 94 cm monitor (1024 × 768 resolution). An experimenter presented the stimuli and recorded data using the Gazetracker^TM^ Image Analysis software. To reduce movement and keep infants secure, they sat in an age-appropriate highchair approximately 61 cm from the eye tracker and 71 cm from the monitor.

**Figure 1:**
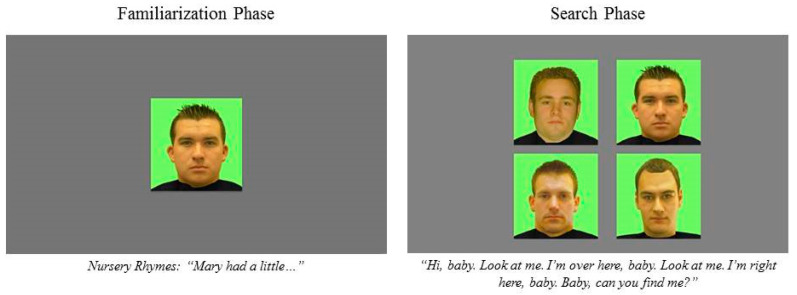
Sample stimuli from a neutral facial expression White male trial.

**Stimuli.** The faces ranged in age from 18–31 years. For the visual search task, there were 40 color familiarization videos of individuals (10 Black women, 10 Black men, 10 White women, 10 White men) speaking nursery rhymes (Mary had a Little Lamb, Jack and Jill and Rub-A-Dub-Dub), 40 static color images of the familiarized faces and 120 static color images of distractor faces (30 Black women, 30 Black men, 30 White women, 30 White men) for each of the conditions. For the neutral condition, we asked models to pose a pleasant but closed mouth neutral expression for the static images and to speak in a calm, neutral tone for the videos. For the pleasant condition, we asked models to pose smiling expressions (up-turned lips) with no teeth showing for the static images and to speak in a pleasant tone as if reading to a child for the videos. The voices were always those of the person shown in the video. We purposely had facial expressions and tone of voice match in affect so the two would not conflict and confuse infants. See Figure 1 for an example of the stimuli.

**Procedure.** Approximately one week before their infant participated in the study, parents came into the lab and a research assistant explained how to complete the surveys. A research assistant followed up with a phone call a couple days later to check on the status of the survey completion and answer any questions parents had. When parents returned with their infant to participate in the visual search task, they received a USD 25 gift card for completing the surveys.

Prior to beginning the study, the experimenter calibrated infant eye movements by separately presenting seven different small videos at seven points on the screen (top left, top right, middle left, middle center, middle right, bottom left and bottom right). Before proceeding to testing, the experimenter presented the calibration stimuli again to check the calibration quality. To start the study and in between each trial, an animation of a rubber duck accompanied by a chiming sound appeared in the middle of the monitor to direct infants’ attention toward the screen. Prior to participating in the visual search task, infants viewed four different female–male face pairs (two Black, two White) with each pair presented individually for 10 s to assess their visual preferences, but those data are not reported here. Then, we tested infants’ recognition of four different faces (1 Black woman, 1 Black man, 1 White woman, 1 White man) following training that taught infants to locate the familiarized face (rather than look at the novel faces) during the visual search. For each trial, infants viewed and heard the familiarization video for 15 s (they heard about 1.5 nursery rhymes). They then saw a static image of the familiarized face presented with three other faces of the same gender, race, age, hair color and attractiveness with faces presented at the top left, top right, bottom left and bottom right of the screen. While these faces were displayed, audio of the familiarized voice played, “Hi baby, look at me. I’m over here, baby. Look at me. I’m right here, baby. Baby, can you find me?” The voice recording continued until the infant located the face and consecutively scanned it for 800 ms [2]. If infants met this criterion, a 5 s reward video played that consisted of the familiarized face stating, “Good job, you found me!” followed by a display of an animated creature or object accompanied by celebratory sounds. If the infant did not locate the familiarized face, the trial ended after 15 s. To train infants to locate the familiarized face and learn they would be rewarded for locating it, they were first familiarized to a different Muppet face on five different trials. During training for the visual search, on the first trial, only the familiarized face was displayed. On the second trial, the familiarized face and one distractor face were displayed. On the third trial, the familiarized face and two distractor faces were displayed. On the fourth and fifth trials, the familiarized face and three distractor faces were displayed. When infants scanned the familiarized face sequentially for 800 ms, they were rewarded. If they did not locate the familiarized face, the training trial repeated one time. 

To ensure generalizability of results, we created ten different orders for each condition in which we varied the familiarization videos and test faces shown in the visual search task. For all orders, we alternated the location of the familiarized face for the visual search. We randomly assigned infants to a condition and order while also attempting to maintain a relatively equal number of infants from each age group in a particular condition and order.

**Figure 2:**
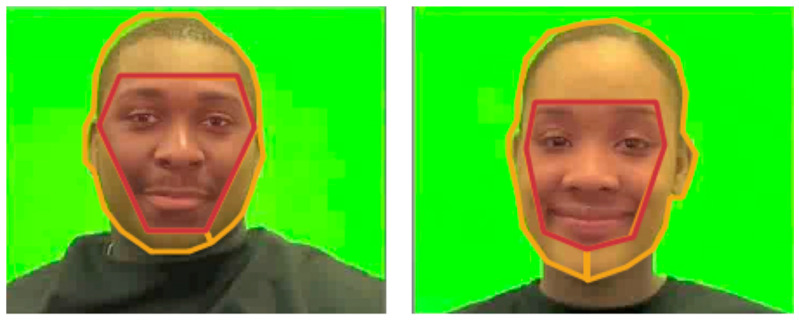
Examples of the internal and external areas-of-interest (AOIs) for two of the faces in the pleasant condition. Note that the AOIs were uniquely adapted to account for movement in the face while the models spoke.

**Data extraction.** To analyze the eye-tracking data, we used Applied Science Laboratories Results Plus (2011) to draw areas-of-interest (AOIs) around the stimuli and extract data for analysis. For the familiarization phase, we created an internal AOI (which included the eyebrows, eyes, nose and mouth) and an external AOI (which included the rest of the face, such as the hair, forehead, ears and chin) for each face (see Figure 2 for examples). For the search phase, we created four box AOIs around each of the three distractor faces and familiarized face. We extracted cumulative dwell data toward AOIs to calculate total looking time during each trial and extracted cumulative fixations toward AOIs to calculate total fixations for each trial. To calculate fixation shifts, we extracted sequential fixation data for each familiarization phase and categorized shifts within internal AOIs and between internal and external AOIs.

## 3. Results

### 3.1. Facial Experience

Based on the IIIS and ICFMIS data that parents provided [9], infants typically had a high percentage of experience with people who were female (*M* = 0.68, *SD* = 0.14, *range* = 0.32–1.00). Their percentage of experience for each of the face types utilized in the visual search task and for face types not utilized in the study are shown in Table 1. The sample’s substantially greater experience with White than Black faces suggests they should have expertise in processing White faces.

### 3.2. Temperament

Infants’ orienting and surgency were positively correlated, *r* = 0.256, *p* = 0.008. For the analyses, we coded infants as high or low in surgency and as high or low in orienting based on a median split of those data [19]. See Table 1 for the means and standard deviations of infants’ surgency and orienting for each of these quadrants.

### 3.3. Visual Search Task

**Overview.** Based on our inclusion criterion (as noted in the Participants section), we had 337 useable trials for analyses. While viewing the familiarization video, infants spent the greatest percentage of their time looking at the face relative to their total looking toward the screen (*M* = 0.83, *SE* = 0.01). While looking at the face, they spent the greatest percentage of their time looking toward the internal facial features (*M* = 0.72, *SE* = 0.02). Despite this attention, they were successful at locating a face only 43% of the time (*SE* = 0.03). Those who were successful at locating the face varied in how long it took them to do so (*M* = 6.73, *SE* = 0.34, *range* = 0.88–12.93 s). During training, 75% of the infants located three or more faces overall (*M* = 3.31, *SE* = 0.07) and typically located at least one face during the last two training trials (*M* = 1.06, *SE* = 0.04), suggesting they learned the task. Indeed, locating a face during visual search positively correlated with the number of training faces located, *rho* = 0.23, *p* < 0.0001. Due to a computer error, an 8-month-old and 10-month-old included in the analyses did not see the training trials. These infants, however, successfully located four or three faces, respectively, during the visual search suggesting the task can be completed without training at these ages. For all useable test trials, we coded whether or not infants successfully located the face and if they located the face, we indicated the time it took to locate it.

**Familiarization.** For each of the dependent variables, we conducted a 2 × 2 × 2 × 2 × 2 × 2 [Condition (neutral, pleasant expression) × Face Gender (female, male) × Face Race (Black, White) × Infant Gender (female, male) × Orienting (high, low) × Surgency (high, low)] linear analysis with repeated measures and infant age in days as a covariate. Face gender and face race were within subject variables and the other variables were between subjects. We included infant gender because research suggests there might be gender differences in scanning behaviors and face recognition abilities [38,43,44]. Only results for the highest order interactions are reported. When decomposing interactions, we utilized the adaptive two-stage procedure to control for both Type I and Type II errors [45]. We report the false discovery rate (FDR) adjusted *p*-value for each relevant analysis. In some cases, the FDR adjusted *p*-value was slightly higher than 0.05. For those instances, we maintained 0.05 as the highest *p*-value for reporting significant results, but still report the FDR adjusted *p*-value. See Table 2 for means, standard errors and ranges for infants’ looking behaviors during familiarization. 

**Figure 3:**
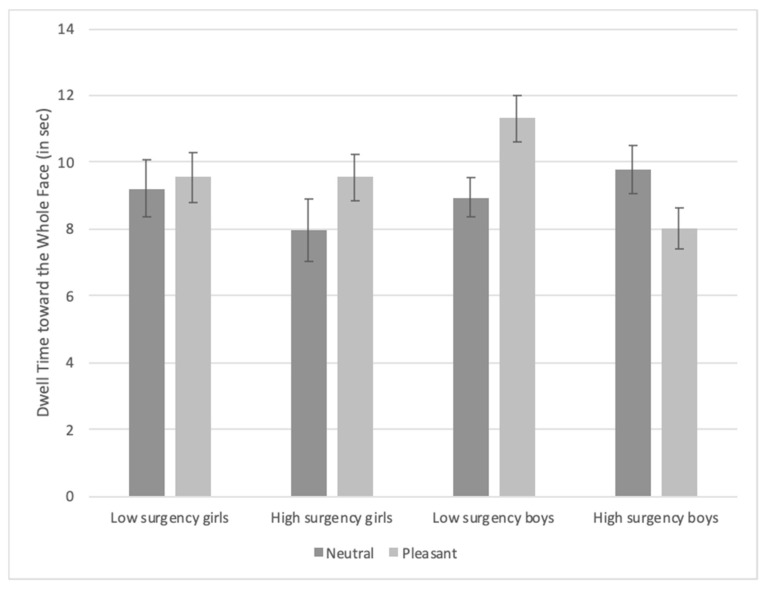
Dwell time toward the familiarized face based on facial expression and infants’ gender and surgency. Bars represent standard error.

***Dwell time toward the whole face.*** There was an Infant Gender × Condition × Surgency interaction, *F*(1, 91) = 6.81, *p* = 0.011, η_p_^2^ = 0.024. The interaction occurred because low-surgency boys viewing pleasant expressions looked longer at the familiarized face compared with high-surgency boys viewing pleasant expressions, *t*(91) = 3.52, *p* = 0.0007, high-surgency girls viewing neutral expressions, *t*(91) = 2.86, *p* = 0.005, and low-surgency boys viewing neutral expressions, *t*(91) = 2.86, *p* = 0.005 [FDR adjusted *p*-value = 0.014] (see Figure 3).

**Figure 4:**
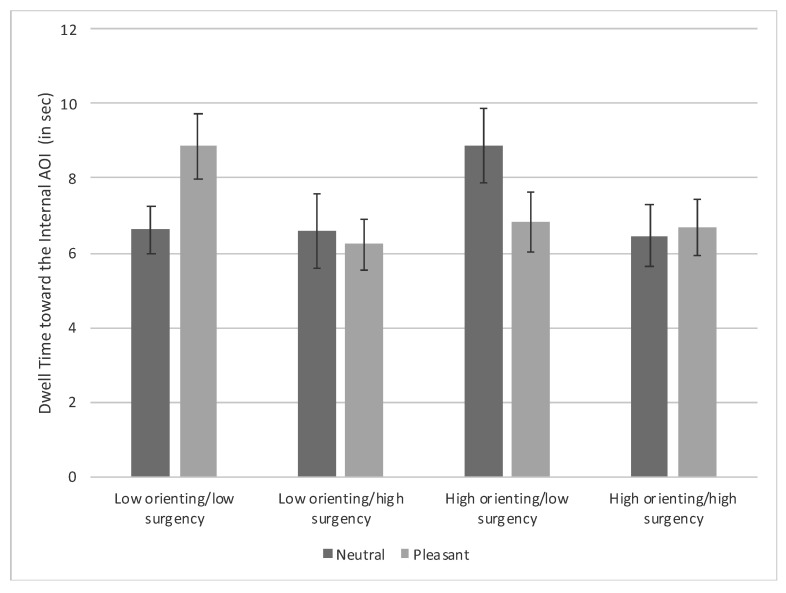
Dwell time toward the internal features of the familiarized face based on facial expression and infants’ orienting and surgency. Bars represent standard error.

***Dwell time toward the internal AOI.*** There was a main effect for surgency, *F*(1, 91) = 4.51, *p* = 0.036, η_p_^2^ = 0.016, that was superseded by a Condition × Orienting × Surgency interaction, *F*(1, 91) = 4.40, *p* = 0.039, η_p_^2^ = 0.016. The interaction occurred because low-orienting/low-surgency infants viewing pleasant expressions looked longer at the internal AOI compared with low-orienting/low-surgency infants viewing neutral expressions, *t*(91) = 2.07, *p* = 0.041, low-orienting/high-surgency infants viewing pleasant expressions, *t*(91) = 2.37, *p* = 0.020, and high-orienting/high-surgency infants viewing neutral expressions, *t*(91) = 2.01, *p* = 0.047. Moreover, high-orienting/low-surgency infants viewing neutral expressions looked longer at the internal AOI compared with low-orienting/high-surgency infants viewing pleasant expressions, *t*(91) = 2.12, *p* = 0.037 [FDR adjusted *p*-value = 0.053] (see Figure 4).

***Dwell time toward the external AOI.*** Only the infant age covariate was significant, *F*(1, 91) = 6.35, *p* = 0.014, η_p_^2^ = 0.023. Increased age in days was related to less looking toward the external facial features, *r* = −0.142, *p* = 0.009. 

**Figure 5:**
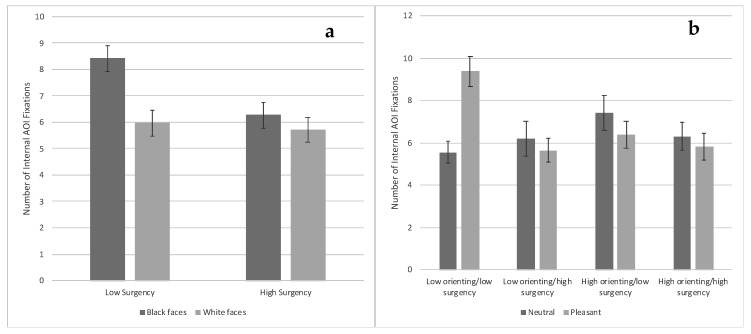
Number of fixations toward the internal features of the familiarized face based on face race and infants’ surgency ((**a**)—left chart) and based on facial expression and infants’ orienting and surgency ((**b**)—right chart). Bars represent standard error.

***Internal AOI fixations.*** There was a main effect of face race, *F*(1, 73) = 9.93, *p* = 0.002, η_p_^2^ = 0.035, and a main effect of surgency, *F*(1, 91) = 5.80, *p* = 0.018, η_p_^2^ = 0.021, that were superseded by a Face Race × Surgency interaction, *F*(1, 73) = 4.02, *p* = 0.049, η_p_^2^ = 0.015. This interaction occurred because low-surgency infants viewing Black faces made significantly more fixations toward the internal AOI compared with high-surgency infants viewing Black faces and both low- and high-surgency infants viewing White faces, *t*s ≥ 3.12, *p*s < 0.003 [FDR adjusted *p*-value = 0.005] (see Figure 5a).

There was also a Condition × Orienting interaction, *F*(1, 91) = 6.42, *p* = 0.013, η_p_^2^ = 0.023, and a Condition × Surgency interaction, *F*(1, 91) = 4.06, *p* = 0.047, η_p_^2^ = 0.015, that were superseded by a Condition × Orienting × Surgency interaction, *F*(1, 91) = 6.75, *p* = 0.011, η_p_^2^ = 0.024. Low-orienting/low-surgency infants viewing pleasant expressions made significantly more fixations to the internal AOI compared with all other infants viewing pleasant expressions, *t*s ≥ 3.14, *p*s < 0.003, and compared with most infants viewing neutral expressions, *t*s ≥ 2.96, *p*s < 0.004, except for high-orienting/low-surgency infants [FDR adjusted *p*-value = 0.005] (see Figure 5b).

**Figure 6:**
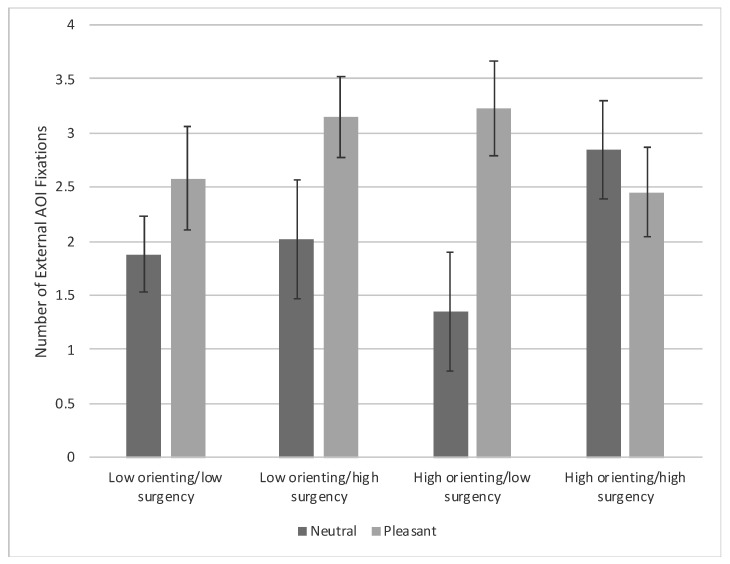
Number of fixations toward the external features of the familiarized face based on facial expression and infants’ orienting and surgency. Bars represent standard error.

***External AOI fixations.*** There was a main effect of condition, *F*(1, 91) = 6.66, *p* = 0.012, η_p_^2^ = 0.024, that was superseded by a Condition × Orienting × Surgency interaction, *F*(1, 91) = 4.45, *p* = 0.038, η_p_^2^ = 0.016. This interaction occurred because low-orienting/high-surgency infants and high-orienting/low-surgency infants viewing pleasant expression faces made significantly more fixations toward the external AOI of the familiarized faces compared with low-orienting/low-surgency infants and high-orienting/low-surgency infants viewing neutral expressions, *t*s ≥ 2.42, *p*s < 0.018. For infants viewing neutral expressions, high-orienting/high-surgency infants made significantly more fixations toward the external AOI of the familiarized faces compared with high-orienting/low-surgency infants, *t*(91) = 2.08, *p* = 0.041 [FDR adjusted *p*-value = 0.048] (see Figure 6). The age covariate was also significant, *F*(1, 91) = 5.89, *p* = 0.017, η_p_^2^ = 0.021, due to a decrease in external AOI fixations with age, *r* = −0.106, *p* = 0.05.

***Internal-internal fixation shifts.*** There was a main effect of face race, *F*(1, 73) = 9.10, *p* = 0.004, η_p_^2^ = 0.032, which occurred because infants made more fixation shifts between the internal facial features of Black faces (*M* = 4.59, *SE* = 0.29) compared with White faces (*M* = 3.33, *SE* = 0.30). 

**Figure 7:**
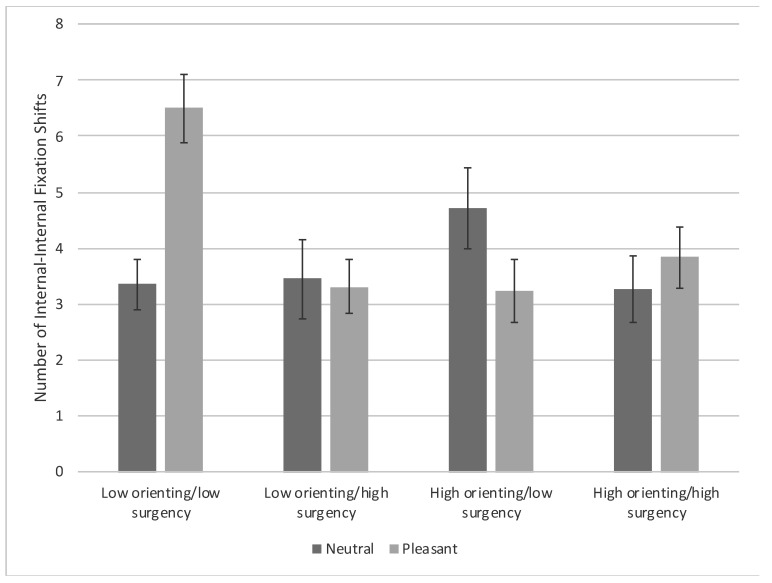
Number of fixation shifts between the internal features of the familiarized face based on facial expression and infants’ orienting and surgency. Bars represent standard error.

There was also a main effect of surgency, *F*(1, 91) = 5.15, *p* = 0.026, η_p_^2^ = 0.019, and an Orienting × Condition interaction, *F*(1, 91) = 5.57, *p* = 0.020, η_p_^2^ = 0.020, that were superseded by an Orienting × Surgency × Condition interaction, *F*(1, 91) = 10.21, *p* = 0.002, η_p_^2^ = 0.036. The interaction occurred because low-orienting/low-surgency infants viewing pleasant expressions made significantly more internal–internal fixation shifts compared with all other infants viewing pleasant expressions, *t*s ≥ 3.26, *p*s ≤ 0.0016. Low-orienting/low-surgency infants viewing pleasant expressions also made significantly more internal–internal fixation shifts compared with low-orienting/low-surgency infants, low-orienting/high-surgency infants and high-orienting/high-surgency infants viewing neutral expressions, *t*s ≥ 3.24, *p*s ≤ 0.0017 [FDR adjusted *p*-value = 0.002] (see Figure 7).

**Figure 8:**
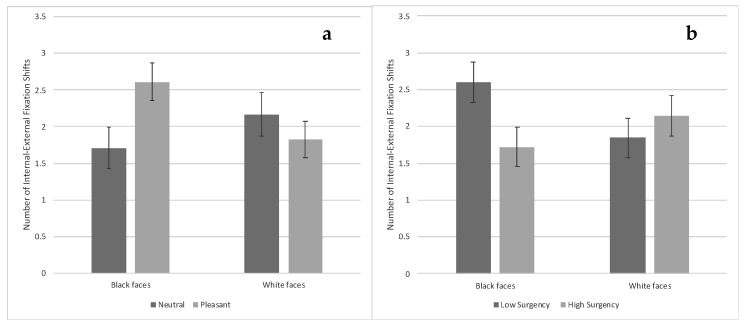
Number of fixation shifts between the internal and external features of the familiarized face based on face race and expression ((**a**)—left chart) and based on face race and infants’ surgency ((**b**)—right chart). Bars represent standard error.

***Internal-external fixation shifts.*** There was a Face Race × Condition interaction, *F*(1, 73) = 5.37, *p* = 0.023, η_p_^2^ = 0.019. Infants viewing Black faces with pleasant expressions made more internal–external fixation shifts compared with infants viewing Black faces with neutral expressions, *t*(73) = 2.38, *p* = *0*.020, and infants viewing White faces with pleasant expressions, *t*(73) = 2.22, *p* = 0.029 [FDR adjusted *p*-value = 0.043] (see Figure 8a). There was also a Face Race × Surgency interaction, *F*(1, 73) = 4.80, *p* = 0.032, η_p_^2^ = 0.017. Low-surgency infants viewing Black faces made more internal–external fixation shifts compared with high-surgency infants viewing Black faces, *t*(73) = 2.22, *p* = 0.029 [FDR adjusted *p*-value = 0.031] (see Figure 8b).

**Figure 9:**
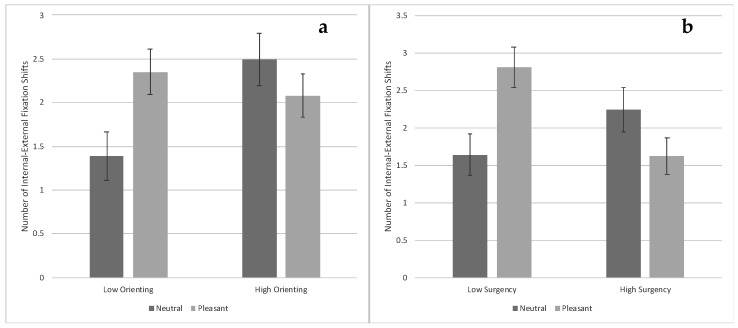
Number of fixation shifts between the internal and external features of the familiarized face based on facial expression and infants’ orienting ((**a**)—left chart) and based on facial expression and infants’ surgency ((**b**)—right chart). Bars represent standard error.

There was also an Orienting × Condition interaction, *F*(1, 91) = 6.50, *p* = 0.012, η_p_^2^ = 0.023. Low-orienting infants viewing neutral expressions made significantly fewer internal–external fixation shifts compared with low-orienting infants viewing pleasant expressions, *t*(73) = 2.56, *p* = 0.012, and high-orienting infants viewing neutral expressions, *t*(73) = 2.73, *p* = 0.008 [FDR adjusted *p*-value = 0.018] (see Figure 9a). In addition, there was a Surgency × Condition interaction, (*F*1, 91) = 11.04, *p* = 0.001, η_p_^2^ = 0.039. Low-surgency infants viewing pleasant expressions made significantly more internal–external fixation shifts compared with low-surgency infants viewing neutral expressions, *t*(91) = 3.07, *p* = 0.003, and high-surgency infants viewing pleasant expressions, *t*(91) = 3.25, *p* = 0.002 [FDR adjusted *p*-value = 0.004] (see Figure 9b).

**Visual search.** To assess infants’ ability to locate the face, a preliminary logistic regression analysis showed that the model did not adequately converge utilizing all six variables included in the familiarization analyses, making the model fit unreliable. We therefore examined the fit of six different models using Aikake’s information criterion (AIC) in which a different variable was excluded in each model. Note that the logistic regression analysis produces only AIC, whereas linear analyses produce both AIC and Aikake’s information criterion corrected (AICC). Therefore, we assessed model fit using AIC for this analysis but used AICC for assessing model fit of our linear analyses. Results showed the best fit to the data excluded infant orienting. We therefore conducted a 2 × 2 × 2 × 2 × 2 [Condition (neutral, pleasant expression) × Face Gender (female, male) × Face Race (Black, White) × Infant Gender (female, male) × Surgency (high, low)] logistic regression analysis with infant age in days as a covariate to assess infants’ ability to locate the face. For these analyses, we also compared infants’ ability to locate a face to a chance rate of 25%.

To assess time to locate the face, we conducted 2 × 2 × 2 × 2 × 2 [Condition (neutral, pleasant expression) × Face Race (Black, White) × Infant Gender (female, male) × Orienting (high, low) × Surgency (high, low)] linear analyses with repeated measures and infant age in days as a covariate. Although the model converged with all six variables from the familiarization analyses included, there was not sufficient power to test some interactions because infants located only 43% of the faces. We chose this model by examining the fit of six different models using AICC in which one of the six variables was excluded in each model. The best fit to the data excluded face gender.

***Ability to locate the face.*** There was a main effect of infant gender, χ^2^ = 5.02, *p* = 0.025. Boys located a greater percent (65.73%) of the faces than girls (34.27%), χ^2^ (1, *N* = 143) = 14.16, *p* = 0.0002. There were more boys than girls in the sample, however, so we compared the percent of faces they each located to chance. Both boys and girls were above chance in locating the faces they saw, but boys had more success (*M* = 46.77%, *SE* = 3.53%), χ^2^ (1, *N* = 201) = 50.79, *p* < 0.0001, than girls (*M* = 36.30%, *SE* = 4.15%), χ^2^ (1, *N* = 135) = 9.19, *p* = 0.002.

**Figure 10:**
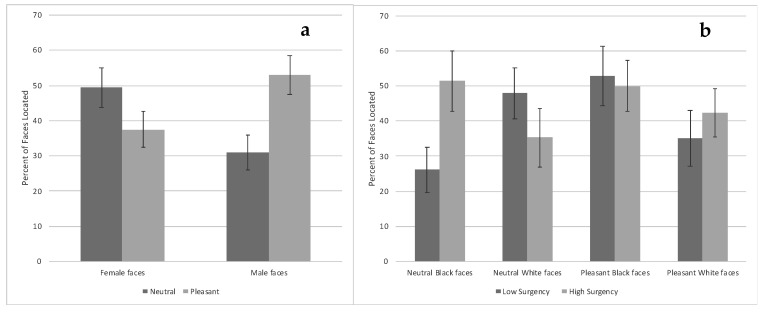
The percent of faces infants located based on face gender and expression ((**a**)—left chart) and their surgency and face race and expression ((**b**)—right chart). Bars represent standard error.

There was also a Condition × Face Gender interaction, χ^2^ = 5.72, *p* = 0.017. Infants located a greater percent of pleasant expression male faces than pleasant expression female faces and neutral expression male faces. Infants also located a greater percent of neutral expression female faces than neutral expression male faces, χ^2^ (1, *N* = 143) = 4.44, *p* = 0.035 (Figure 10a). Infants were above chance in locating female faces regardless of whether they had neutral expressions, χ^2^ (1, *N* = 79) = 25.02, *p* < 0.0001, or pleasant expressions, χ^2^ (1, *N* = 88) = 7.33, *p* = 0.007. In contrast, infants were at chance in locating neutral expression male faces, χ^2^ (1, *N* = 84) = 1.59, *n.s.*, and above chance in locating pleasant expression male faces, χ^2^ (1, *N* = 85) = 35.39, *p* < 0.0001.

There was also a Condition × Face Race × Surgency interaction, χ^2^ = 6.43, *p* = 0.011. Comparisons among low-surgency infants showed they located a lower percent of neutral expression Black faces compared with neutral expression White faces and pleasant expression Black faces. Comparisons between high- and low-surgency infants showed that high-surgency infants located a greater percent of neutral Black faces compared with low-surgency infants. Moreover, high-surgency infants located a greater percent of pleasant Black faces and pleasant White faces compared with low-surgency infants locating neutral expression Black faces (Figure 10b). Low-surgency infants were at chance locating neutral expression Black faces, χ^2^ (1, *N* = 46) = 0.03, *n.s.*, and pleasant expression White faces χ^2^ (1, *N* = 37) = 2.03, *n.s.*, but were above chance in locating neutral expression White faces, χ^2^ (1, *N* = 48) = 13.44, *p* < 0.0001, and pleasant expression Black faces, χ^2^ (1, *N* = 36) = 14.82, *p* < 0.0001. High-surgency infants were at chance locating neutral expression White faces, χ^2^ (1, *N* = 34) = 1.92, *n.s.*, but were above chance at locating neutral expression Black faces, χ^2^ (1, *N* = 35) = 13.04, *p* < 0.0001, pleasant expression Black faces, χ^2^ (1, *N* = 48) = 16.00, *p* < 0.0001, and pleasant expression White faces, χ^2^ (1, *N* = 52) = 8.31, *p* = 0.004.

We also conducted a supplementary analysis with infant orienting included and face gender excluded, so the variables were the same as those in the time to locate the face analysis (described below). Similar to the analysis including face gender and excluding infant orienting, results showed a main effect of infant gender, χ^2^ = 5.20, *p* = 0.023, and a Condition × Face Race × Surgency interaction, χ^2^ = 4.82, *p* = 0.028. The main effect and interaction were superseded by an Infant Gender × Face Race × Orienting × Surgency interaction, χ^2^ = 3.85, *p* = 0.0497. There was not sufficient power to decompose the four-way interaction because some of the cell sizes were as low as 3.

**Figure 11:**
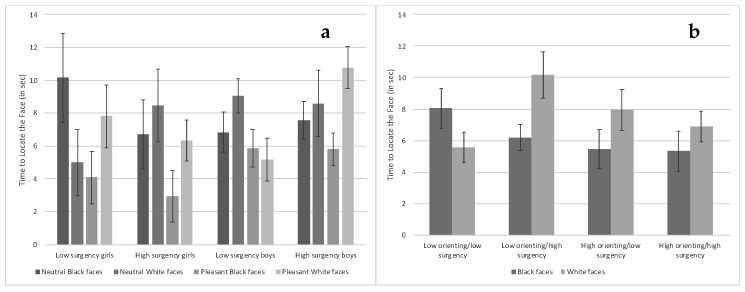
The time it took to locate faces based on infant surgency and gender and face race and expression ((**a**)—left chart) and based on infant orienting and surgency and face race ((**b**)—right chart). Bars represent standard error.

***Time to locate the face***. There was a main effect of condition, *F*(1, 52) = 4.12, *p* = 0.047, η_p_^2^ = 0.036, which was superseded by an Infant Gender × Condition × Face Race × Surgency interaction, *F*(1, 23) = 4.50, *p* = 0.045, η_p_^2^ = 0.039. Low-surgency boys were significantly quicker at locating pleasant expression Black faces and pleasant expression White faces compared with neutral expression White faces, *t*s ≥ 2.11, *p*s < 0.046. High-surgency boys were quicker at locating pleasant expression Black faces compared with pleasant expression White faces, *t*(23) = 3.09, *p* = 0.005. Last, high-surgency girls and low-surgency boys were significantly quicker at locating pleasant expression White faces compared with high-surgency boys, *t*s ≥ 2.50, *p*s < 0.020 [FDR adjusted *p*-value = 0.05] (Figure 11a).

There was also a Face Race × Orienting × Surgency interaction, *F*(1, 23) = 5.04, *p* = 0.035, η_p_^2^ = 0.044. This interaction occurred because low-orienting/high-surgency infants took more time to locate White faces than Black faces, *t*(23) = 2.40, *p* = 0.025. Low-orienting/high-surgency infants also took longer to locate White faces compared with low-orienting/low-surgency infants locating White faces and high-orienting/low-surgency and high-orienting/high-surgency infants locating Black faces, *t*s ≥ 2.43, *p*s < 0.024 [FDR adjusted *p*-value = 0.028] (Figure 11b).

**Relation between behavior during familiarization and visual search performance.** We conducted correlations between the familiarization variables and ability and time to locate the face. Dwell time to the whole face, internal facial features and external facial features and number of internal–external fixation shifts were all positively correlated with ability to locate the face, whereas number of internal or external facial feature fixations and internal–internal fixation shifts were unrelated to locating the face. Only dwell time to the whole face and internal facial features were negatively correlated with time to locate the face (Table 3).

**Role of temperament.** The linear models utilized in this study were complex and involved four between-subjects variables and one or two within-subjects variables with results often producing high-order interactions. Subsequently, we assessed whether including the two temperament measures provided a better fit to the data than not including those measures by comparing the AICC for the two different models. A lower AICC value indicates a better fit. For all linear analyses, including the temperament measures provided a better fit to the data (Table 4). In contrast, the logistic regression analysis assessing ability to locate the face showed the best fit with infant orienting excluded (as noted previously).

## 4. Discussion

The main goal of this research was to examine whether individual differences in infant temperament interacted with facial characteristics to predict their looking behavior during familiarization and ability and time to locate familiarized faces. For eight of the nine dependent variables analyzed, including the infant surgency and orienting variables improved the fit of the model, so temperament appears to be a critical factor for understanding infants’ face processing and recognition. We expected high-orienting infants would display scanning behaviors during familiarization that correlated with locating a higher number of faces in a quicker period of time, but we were uncertain as to whether high- or low-surgency would function in a similar manner. Results showed that infants’ surgency regularly predicted their face processing and recognition abilities, whereas infant orienting showed less consistency as a predictor variable. Interestingly, infants’ surgency and/or orienting often interacted with facial expression or race, but not face gender, to predict their scanning behaviors during familiarization and location of faces. Face gender, however, did interact with facial expression to facilitate face recognition as predicted, such that infants were more successful at locating pleasant than neutral expression male faces. Findings demonstrate the interplay between how infant traits and target characteristics affect face processing.

We expected infant temperament would predict behaviors during familiarization trials related to their face recognition abilities. Behaviors during familiarization that positively predicted locating the face included dwell time toward the whole face and internal and external facial features, as well as number of internal–external fixation shifts. Dwell time to the whole face and internal facial features also negatively predicted location time. One commonality for these variables (whole face dwell time, internal AOI dwell time and internal–external fixation shifts) is that low surgency interacted with facial characteristics, and sometimes orienting, to predict longer dwell times and more internal–external fixation shifts. For example, low-surgency boys viewing pleasant expression faces had the longest whole face dwell time. Low-orienting/low-surgency infants viewing pleasant expressions and high-orienting/low-surgency infants viewing neutral expressions had the longest internal AOI dwell times. Moreover, low-surgency infants made more internal–external fixation shifts when viewing Black faces or pleasant expression faces compared with high-surgency infants. Our findings of a link between infants’ low surgency and attention is similar to research showing that the longer 4- to 10-month-olds’ fixation durations were toward dynamic faces, shapes and objects, the lower their surgency was at ~3.5 years of age [26]. Compared with high-surgency infants, low-surgency infants have lower activity levels and impulsivity [24], which might explain their longer viewing times and greater number of fixation shifts—they should be able to stay still and focus longer on the stimuli. Such differences might be particularly evident when infants are viewing unfamiliar race (Black) faces and pleasant expression faces—compared with low-surgency infants, high-surgency infants typically experience more intense pleasure when viewing stimuli that are novel or conveying a positive emotion indicative of a potential social interaction [24]. This greater level of intensity could interfere with high-surgency infants’ ability to regulate their emotions [25] and subsequently affect their attention during looking time studies more so than low-surgency infants.

Results also showed that our hypothesis regarding infant orienting was too simplistic. We expected high orienting would predict scanning behaviors during familiarization that correlated with ability and time to locate the face. Instead, infants’ orienting interacted with surgency to differentially affect their attention toward neutral and pleasant expression faces during familiarization. For example, dwell times toward internal facial features were highest among low-orienting/low-surgency infants viewing pleasant expressions and high-orienting/low-surgency infants viewing neutral expressions. High orienting is related to experiencing pleasure from stimuli low in intensity and ability to maintain attention, whereas low orienting is related to feeling pleasure from stimuli high in intensity [24], so that could explain these findings. Infants’ low surgency likely facilitated maintenance of attention toward the internal facial features, but low-orienting infants were more drawn to the pleasant expression (higher intensity) faces and high-orienting infants were more drawn to the neutral expression (lower intensity) faces. A similar pattern was seen in these infants’ internal–external fixation shifts—low-orienting infants viewing pleasant expressions and high-orienting infants viewing neutral expression made more internal–external fixation shifts compared with low-orienting infants viewing neutral expressions. Both the infant surgency and orienting results show that racial novelty and expression intensity of the faces interacted with infant temperament to affect attention.

Surgency and orienting also interacted to predict fixations toward internal and external facial features as well as fixation shifts between internal facial features, but these behaviors during familiarization did not significantly correlate with infants’ ability or time to locate the faces during visual search. Nonetheless, number of internal fixations and internal–internal fixation shifts positively correlated with dwell times toward the whole face and internal facial features (*r*s > 0.51, *p*s < 0.0001) and number of external fixations positively correlated with dwell times toward the external facial features (*r* = 0.672, *p* < 0.0001). These correlations thereby suggest number of fixations and internal–internal fixation shifts had an indirect effect on infants’ face recognition. Therefore, although we did not replicate previous research showing shifts between internal facial features were directly correlated to discriminating between familiar and novel faces [39,40], it is plausible that such shifts indirectly contributed to face recognition abilities in the current study by increasing dwell time. Dissimilarities between our study and these previous studies could also be due to differences in how we tested face recognition. Following habituation or familiarization in the previous studies, infants saw a novel and familiar face paired together and longer looking toward the novel than familiar face indicated face discrimination [39,40]. In our study, infants saw a familiar and three novel faces displayed together with the familiarized voice encouraging infants to locate and scan the familiar face. Such discrepancies during the test trials might require slightly different scanning strategies during familiarization. Perhaps internal–external fixation shifts during familiarization better facilitate face location among a set of faces compared with internal–internal fixation shifts.

Some evidence to support the possibility that various scanning strategies might be more effective for certain tasks than others comes from our finding that longer dwell times to the whole face showed the strongest correlations with ability to locate the face and quicker location times. Interestingly, we found longer looking during familiarization correlated with face recognition, whereas Quinn et al. did not [32]. Again, the differences could be due to our using visual search to locate a familiarized face and Quinn et al. using a novelty preference to assess face recognition [32]. Moreover, our familiarized faces were dynamic and speaking nursery rhymes, whereas Quinn et al.’s faces were static and there was no speech associated with the faces [32]—those stimulus differences could potentially impact how infants’ scanned the faces during familiarization. More research is needed to understand how both characteristics of the faces during familiarization trials and the method for testing face recognition affect looking behaviors during familiarization and the behaviors that best predict face recognition.

Despite our finding a correlation between familiarization dwell time and face recognition whereas Quinn et al. did not, we did replicate their finding that pleasant expression facilitates recognition of unfamiliar race faces [32] with the caveat that it depended on infant surgency. Infants in our study were about twice as successful at locating pleasant expression Black faces compared with neutral expression Black faces if they were low in surgency. In contrast, high-surgency infants were equally successful at locating neutral and pleasant expression Black faces. Quinn et al. posited that infants’ better recognition of pleasant than neutral expression unfamiliar race faces might be due to deeper processing of the pleasant faces [32]. Some data to support this possibility are that infants in the current study made significantly more internal–external fixation shifts (potential indicators of holistic processing) when viewing pleasant relative to neutral expression Black faces. Furthermore, low-surgency infants made significantly more internal–external fixation shifts toward Black faces and toward pleasant expressions compared with high-surgency infants. Internal–external fixation shifts should facilitate perceiving the face as a whole rather than as separate components of features [37]. Holistic processing facilitates adults’ encoding of emotional expressions [46] and face recognition [47]. If encoding pleasant emotional expressions also facilitates holistic processing among infants, this deeper processing might also improve recognition of unfamiliar race faces [48].

Although temperament interacted with face race and expression to influence infant eye movements, it did not interact with face gender to affect scanning behaviors during familiarization. Moreover, face gender did not interact with facial expression to affect scanning behaviors during familiarization. Infants in our sample experienced male faces less frequently than female faces, but the disparities were not nearly as large as the discrepancies in their experience with White and Black faces. As such, face gender had less impact on infant behaviors during familiarization compared with face race. Infants’ ability to locate female and male faces during visual search, however, did vary as a function of facial expression as we hypothesized. Infants were significantly more successful at locating pleasant than neutral expression male faces—indeed, they were below chance at locating neutral expression males. In contrast, they were above chance in locating female faces regardless of expression. As hypothesized, infants’ predominant experience with women and their various facial expressions likely contributed to similar success in locating neutral and pleasant expression female faces. For male faces, however, the pleasant expressions might have facilitated holistic processing and subsequent face location, similar to our explanation for improved recognition of pleasant expression Black faces.

Similar to previous research [43,44], boys showed an advantage in face recognition. Yet, infant gender predicted only one of the familiarization behaviors—low-surgency boys showed long dwell times toward pleasant expression faces during familiarization. Dwell time was a significant predictor of both ability and time to locate the face. Indeed, low-surgency boys were significantly quicker at locating pleasant expression faces (both Black and White) compared with neutral White faces. Number of internal–external fixation shifts was also a significant predictor of ability to locate a face and prior work found that percentage of internal–external fixation shifts was higher among male infants and adults than female infants and adults [38]. There were no main effects or interactions involving infant gender in predicting internal–external fixation shifts in the current study though, possibly because our faces were dynamic, whereas Rennels and Cummings’ faces were static [38]. A review of the current study’s raw totals showing infants’ success at locating a face or not based on surgency, condition and infant gender showed only low-surgency boys viewing pleasant expressions located more faces than not. The second most successful group in locating faces was high-surgency boys viewing pleasant expressions. These data suggest pleasant expressions might have facilitated face processing more so for boys than girls. Possible reasons for this gender difference could be related to male infants being more receptive to the novel facial stimuli utilized in the study compared with female infants [49] and/or male infants’ typically greater enjoyment when viewing high-intensity stimuli (i.e., pleasant expressions) relative to female infants [50]. More research is needed to test these possibilities and understand male infants’ advantage in face recognition.

Unlike previous studies, infant age had limited influence on infants’ scanning behavior during familiarization. We found only two correlates with age. Infants’ dwell time and fixations toward the external facial features decreased with age. This finding fits with other research showing that a reliance on external features for processing faces decreases during the second half of the first year [51]. One potential reason why age effects were limited in our study is because previous research reported age differences in attention toward particular internal facial features, e.g., [52,53]. In contrast, we assessed attention toward internal and external facial features in general. Another possibility is that given the improved fit of our models with infant temperament added, it may be that infants’ surgency and orienting more strongly influence their scanning behaviors than age. Reanalyzing these data using separate AOIs for the eyes, nose and mouth would enable a test of these various explanations.

Our findings that infant temperament interacted with stimulus characteristics to affect processing are likely not specific to faces. Rather, temperament is thought to affect infants responding to both social and non-social stimuli [24]. For example, indicators of infants’ surgency (e.g., smiling, positive vocalizations) differentially affected their attention and behavior (latency to approach and grasp a toy) toward novel, low-intensity and high-intensity toys [49]. Moreover, infants’ visual attention toward both objects and faces, as measured by fixation durations, predicted their effortful control and surgency during childhood [26]. Additional research assessing the same infants’ attention toward both faces and objects would illuminate how similarly temperament influences such processing.

A limitation of this research is that the visual search task seemed challenging for the infants—they located only 43% of the faces. Compared with another study utilizing the same task, but with infants aged 10 to 16 months, infants located ~63% of the faces [2]. That study, however, utilized only familiar race female and male faces, so it is unclear whether the differences in successful face location for the two studies are due to infant age or facial characteristics or some combination thereof. Upon reviewing the percentage of faces infants located on the visual search trials in the current study, they had more success on trials 1 and 2 than trials 3 and 4, possibly due to fatigue. Future research utilizing this paradigm with younger infants might want to include only two test trials. Another limitation is that our paradigm utilized familiarization trials that included speech and a visual search task to assess face recognition making it more difficult to compare our results to studies utilizing familiarization or habituation/novelty preference paradigms. Nonetheless, face recognition is considered critical for remembering familiar individuals and knowing how to respond accordingly, so it could be argued that the paradigm for the current study more closely mirrors the importance of locating and identifying familiar people compared with novelty preference paradigms.

## 5. Conclusions

Infants’ surgency on its own or in conjunction with infant orienting affected how they scanned neutral versus pleasant expressions and familiar and unfamiliar race faces. Differences in scanning, such as longer dwell times to the facial features and more internal–external fixation shifts, directly correlated with infants’ ability to locate the familiarized face. Dwell times to the whole face and internal facial features also predicted how quickly they could locate the face. In real-world contexts, such behaviors are critical for locating a caregiver in a timely manner. Given the relative stability in individuals’ surgency between infancy and 4 years of age [24], these face processing abilities might maintain during these periods when children are highly dependent on caregivers. Assessing the stability of face discrimination abilities is an important direction for future research. Moreover, investigating more about how surgency impacts cognitive and emotional regulation during early development could be useful for understanding ways to improve face processing. Last, findings hint at the possibility that poorer recognition of other-race individuals and male faces is not inevitable if exposed to those individuals during positive interactions. Assessing how much exposure is necessary to overcome these typical effects remains to be answered in future research.

## Figures and Tables

**Table 1:** Means, standard deviations and ranges for infants’ percent of experience with faces based on gender and race and for the temperament measures.

Average Percent Facial Experience	Temperament
White females (*M* = 0.42, *SD* = 0.29; *range* = 0–0.94) White males (*M* = 0.20, *SD* = 0.15; *range* = 0–0.56) Black females (*M* = 0.05, *SD* = 0.15; *range* = 0–0.91) Black males (*M* = 0.02; *SD* = 0.05; *range* = 0–0.32) Other race females (*M* = 0.21, *SD* = 0.24; *range* = 0–0.84) Other race males (*M* = 0.10, *SE* = 0.14; *range* = 0–0.65)	Low surgency (*M* = 4.43, *SD* = 0.48;), low orienting (*M* = 4.32, *SD* = 0.57) − *n* = 30 Low surgency (*M* = 4.36, *SD* = 0.54), high orienting (*M* = 5.61, *SD* = 0.42) − *n* = 23 High surgency (*M* = 5.45, *SD* = 0.30), low orienting (*M* = 4.56, *SD* = 0.50) − *n* = 25 High surgency (*M* = 5.72; *SD* = 0.42), high orienting (*M* = 5.64, *SD* = 0.40) − *n* = 30

**Table 2:** Infant looking behavior during the familiarization video.

	Whole Face Dwell Time (sec)	Internal AOI Dwell Time (sec)	External AOI Dwell Time (sec)	Internal AOI Fixations	External AOI Fixations	Internal–Internal Fixation Shifts	Internal–External Fixation Shifts
*Mean*	9.07	6.96	2.12	6.33	2.48	3.79	1.01
*Std Error*	0.23	0.26	0.15	0.22	0.14	0.19	0.07
*Range*	1.00–15.02	0.00–15.02	0.00–15.02	0–20	0–18	0–18	0–6

**Table 3:** Correlations between infant looking behavior during familiarization with ability to locate the face (Spearman rho) and time to locate the face (Pearson) overall and adjusted for temperament.

	Whole Face Dwell Time	Internal AOI Dwell Time	External AOI Dwell Time	Internal AOI Fixations	External AOI Fixations	Internal–Internal Fixation Shifts	Internal–External Fixation Shifts
Face located	0.228 ***	0.146 **	0.130 *	0.087	0.078	0.092 †	0.108 *
adjusted for temperament	0.236 ***	0.151 **	0.129 *	0.093†	0.078	0.096 †	0.110 *
Time to locate	−0.255 **	−0.180 *	−0.056	−0.03	0.03	−0.02	−0.135
adjusted for temperament	−0.260 **	−0.186 *	−0.052	−0.1	0.032	−0.032	−0.134

*** *p* < 0.0001, ** *p* < 0.01, * *p* < 0.05, † *p* < 0.10.

**Table 4:** Comparison of the linear models excluding and including infant temperament measures using Akaike’s information criterion corrected (AICC).

Dependent Variable	AICC without Temperament Measures	AICC with Temperament Measures
Dwell time toward whole face	1907.6	1688.1
Dwell time toward internal AOI	1975.0	1754.5
Dwell time toward external AOI	1638.4	1465.3
Fixations toward internal AOI	1866.4	1642.0
Fixations toward external AOI	1588.7	1427.8
Internal-internal fixation shifts	1783.4	1570.0
Internal-external fixation shifts	1508.4	1333.7
Time to locate face	785.4	660.1
